# MYO18A Expression is a Prognostic Factor for Progression-Free Survival in Grade 4 Adult gliomas. Preliminary Report

**DOI:** 10.32604/or.2026.074078

**Published:** 2026-04-22

**Authors:** Aleksander Strąk, Ludmiła Grzybowska-Szatkowska, Paweł Cisek, Marta Ostrowska-Leśko, Jarosław Dudka, Joanna Kubik, Jacek Osuchowski, Paweł Szmygin, Bożena Jarosz, Andrzej Krajka, Tomasz Krajka, Kazimierz Szatkowski, Brygida Ślaska

**Affiliations:** 1 Department of Radiotherapy, Medical University of Lublin, Radziwiłłowska 13, Lublin, Poland; 2 Chair and Department of Toxicology, Medical University of Lublin, Jaczewskiego 8B, Lublin, Poland; 3 Independent Medical Biology Unit, Medical University of Lublin, Jaczewskiego 8B, Lublin, Poland; 4 Chair and Department of Neurosurgery and Pediatric Neurosurgery, Medical University of Lublin, Jaczewskiego 8B, Lublin, Poland; 5 Institute of Computer Science, Maria Curie-Sklodowska University, Lublin, Poland; 6 Division of Mathematics, Department of Production Computerisation and Robotisation, Mechanical Engineering Faculty, Lublin University of Technology, Nadbystrzycka 36, Lublin, Poland; 7 Faculty of Management, Lublin University of Technology, Nadbystrzycka 38D, Lublin, Poland; 8 Institute of Biological Bases of Animal Production, University of Life Sciences in Lublin, Akademicka 13, Lublin, Poland

**Keywords:** MYO18A—myosin-18A, brain gliomas, mRNA, Golgi apparatus

## Abstract

**Objectives:**

Brain gliomas are among the tumors with the worst prognosis, and their incidence is increasing. Postoperative temozolomide-based chemoradiotherapy for grades 3 and 4 gliomas extended overall survival (OS) by approximately two months. An increasing number of clinical trials are investigating molecular-based therapy. Recent studies have demonstrated the involvement of Golgi apparatus proteins, including MYO18A (myosin-18A), in processes associated with abnormal proliferation, migration, apoptosis evasion, and angiogenesis promotion. The aim of this study was to investigate whether MYO18A has prognostic value in patients treated for brain gliomas.

**Methods:**

The research material in the work included tumor samples taken during neurosurgery and blood samples from 45 patients treated for brain gliomas with grade of 1 to 4 according to WHO, which were used to determine the expression of *MYO18A* mRNA (messenger ribonucleic acid). Expression of *MYO18A* was presented as fold changes in RQ (relative quantification) mRNA levels.

**Results:**

This study showed higher *MYO18A* values in patients diagnosed with grade G4 glioma among those with a shorter progression-free survival (PFS) time and those living shorter than the group average. However, statistically significant differences were achieved only for PFS for the *MYO18A* RQ feature (PFS = 4.64, SD = 2.16 vs. PFS = 15.83 and SD = 7.27, *p* = 0.0231). Also, a positive correlation was demonstrated between tumor volume and *MYO18A* expression.

**Conclusion:**

The level of expression of *MYO18A* can be considered a prognostic factor for PFS in patients treated for G4 gliomas, because higher *MYO18A* expression was associated with earlier recurrence.

## 1 Introduction

The most common type of primary malignant brain tumors in adults is gliomas [1,2]. These are tumors originating from glial cells. The most common of them is WHO G4 glioblastoma multiforme (GBM), which occurs with an incidence of 3.23/100,000 people and constitutes approximately 58% of all malignant gliomas. Other gliomas occur less frequently—diffuse astrocytoma—0.46/100,000, anaplastic astrocytoma—0.42/100,000, oligodendroglioma—0.23/100,000, and anaplastic oligodendroglioma—0.11/100,000 [2]. In the Polish population, approximately 2500 new cases of gliomas are diagnosed annually, which constitutes approximately 1.5% of newly diagnosed cancers (data for 2021) [3]. The incidence of brain gliomas is higher in men than in women (5.51/100,000 and 3.65/100,000, respectively), with the exception of diffuse midline gliomas [3,4].

The etiology of brain gliomas is only poorly known. It is believed that their development is caused by genetic changes in glial stem or progenitor cells. Ionizing radiation is a factor that has been proven to increase the risk of disease. Gliomas are more common in people who have had radiotherapy to the head and neck area in the past [4,5]. About 5% of gliomas run in families [5,6], and (1–2)% are hereditary. Li-Fraumeni syndrome, Turcot syndrome, and neurofibromatosis type 1 are associated with the highest risk of developing gliomas [5,6]. Also, the history of a lower-grade glioma is a risk factor for the development of a higher-grade glioma. Allergic and atopic diseases are associated with a 22% reduction in the risk of developing brain tumors, especially gliomas. This is probably due to the stimulation of the immune system, which hinders the neoplastic transformation of glial cells [7,8].

There are conflicting data regarding the relationship between the use of mobile phones and the risk of brain gliomas [9,10]. Some studies indicate an approximately 40% increase in the risk of brain glioma on the side where the phone is most often used [9]. Other studies do not confirm this fact [10].

The Golgi apparatus (GA) is part of the intracellular membrane system, which also includes the smooth and rough endoplasmic reticulum, vacuoles, and lysosomes. The Golgi apparatus (GA) is composed of protein-lipid membranes arranged on top of each other in the shape of elongated vesicles (cysterns). 5 to 8 cisterns with secretory vesicles form the Golgi stack, i.e., the dictyosome [11].

The main functions of GA are to modify proteins and lipids produced in the rough endoplasmic reticulum. This modification may involve the addition of sugar (glycosylation), sulfate (sulfurylation), phosphate (phosphorylation), and lipid (acylation) residues [12–14]. Another example of modification may be the so-called limited proteolysis, i.e., removal of protein fragments that block the activity of proproteins (e.g., proinsulin) [15]. Other GA functions include the final stage of biochemical processing of lipids produced in the smooth endoplasmic reticulum and sorting of proteins and lipids before delivering them to other places in the cell. The Golgi apparatus is also involved in the transmembrane transport of ions and metals [16].

**Figure 1:**
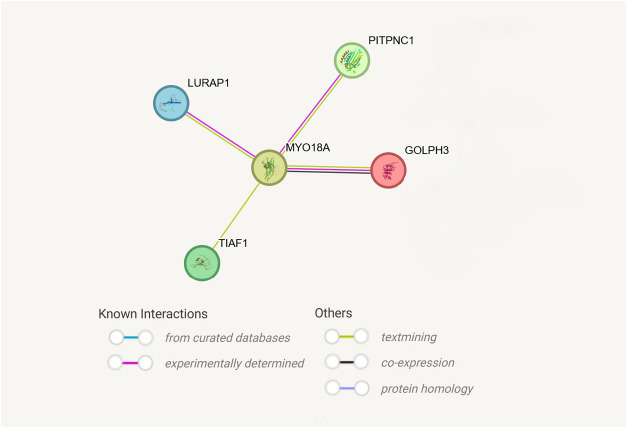
Example of interactions between *MYO18A* and other signalling pathways in the cell in acute myeloid leukemia and types of gene interactions [19]. Figure created using the String database [22]. Abb: *GOLPH3—*Golgi Phosphoprotein 3; *PITPNC1*—phosphatidylinositol transfer protein cytoplasmic 1; *TIAF1—TGFB1*-induced anti-apoptotic factor; *LURAP1*—Leucine rich adaptor protein 1, *MYO18A—*Myosin-18A.

GA is not only a site of protein synthesis, transport, and segregation, but also a signaling node of pathways associated with cancer promotion and progression. It regulates the processes of autophagy, apoptosis, inflammation, DNA repair, and cell polarity [17,18]. Proteins involved in promoting features characteristic of a cancer cell and related to the Golgi apparatus include, among others, myosin 18A (MYO18A) [19–21]. The *MYO18B* is considered a tumor suppressor gene in breast cancers, lung cancers, and ovarian cancers [20–22], but the role of *MYO18A* is not established. Fig. 1 shows an example of interactions between MYO18A and other cellular pathways in the case of acute myeloid leukemia.

MYO18A plays an important role in organizing the cell cytoskeleton through interaction with non-muscle myosin II-A [23]. Some cancers showed a poor prognosis when MPV (mean platelet volume) increased [24]. Changes in platelet morphology influence the coagulation system and the secretion of proinflammatory factors. Myosin IIA, which interacts with MYO18A, may influence platelet size, although a link between MYO18A and MPV has not yet been demonstrated.

The study examined the expression of *MYO18A* at the mRNA (messenger ribonucleic acid) level. The aim of the study was to investigate whether *MYO18A* has a prognostic value in patients treated for brain gliomas. The correlation between *MYO18A* RQ values and the differentiation grade of brain gliomas, as well as the correlations between *MYO18A* and morphological and biochemical blood parameters and clinical factors, were also assessed. An analysis of the survival of patients diagnosed with brain glioma was performed.

## 2 Materials and Methods

### 2.1 Material

The research material included tumor samples taken during neurosurgery and blood samples from 45 patients treated for brain gliomas with malignancy grades 1 to 4 according to WHO at the Department of Neurosurgery and Pediatric Neurosurgery of the Independent Public Clinical Hospital No. 4 in Lublin in the years 2017–2020. Volume of the tumor material collected was 3 mm^3^. Blood samples were taken before the operation.

The macroscopic diagnosis of cancer was then confirmed by histopathological examination. The histopathological diagnosis was made based on the 2021 World Health Organization (WHO) classification of central nervous system tumors [25].

The inclusion criteria for study participants were as follows:

- none of the patients undergoing surgery had received prior oncological treatment, including radiotherapy or chemotherapy,

- all patients had undergone radical macroscopic tumor removal,

- all patients had a postoperative histopathological diagnosis of G1-G4 glioma.

The exclusion criteria for the study were:

- performance status above 2 on the WHO scale

- presence of another cancer that is not being treated

The size of the tumor was assessed manually by MRI (Magnetic Resonance Imaging) scan, and disease progression was also determined by MRI.

The samples of control tissue weretaken from patients who had undergone neurosurgery for reasons other than cancer. Informed consent was obtained from all subjects involved in the study. The study was conducted in accordance with the Declaration of Helsinki, and approved by The Bioethics Committee at the Medical University of Lublin, Av. Racławickie 1, 20-059 Lublin, Poland (bioethics committee approval number KE-0254/171/2017 and KE-0254/74/2019).

### 2.2 Research Methodology

Tumor material and control tissue material collected during surgery were stored in RNAlater^®^ RNA Stabilization Reagent (QIAGAGEN, Hilden, Germany, cat. no. 76106) to inactivate endogenous RNAses and then frozen at −80°C. These samples were used to determine *MYO18A* mRNA expression. Additionally, selected morphological and biochemical parameters were determined from patients’ blood collected before surgery. (Supplementary materials Table S1)

The *MYO18A* variability model was assessed based on morphological and biochemical parameters, as well as patient age and tumour size.

The main source of information about patients operated on for gliomas was disease histories from the Independent Department of Pediatric Neurosurgery and Neurosurgery Public Clinical Hospital No. 4 in Lublin. Information about the further fate of the patients was obtained from patients themselves and their families or by analyzing medical documentation. The patients were divided into two groups depending on the diagnosis according to the WHO. The first group was patients diagnosed with glioma with histological grade G3 and G4 according to WHO, the second group was patients treated for G1 and G2 gliomas. The third group was the control group.

The basic criteria assessed were overall survival (OS) and progression-free survival (PFS). OS is the time after surgery (day of treatment initiation) until the date of death or the date of the last follow-up examination in the observation period, which was conducted until 10 January 2024. PFS was counted from the start of treatment to the date of disease progression, as described in the imaging study.

The results were assessed using statistical methods.

### 2.3 Methods

#### 2.3.1 RNA Isolation

*MYO18A* gene expression at the mRNA level was determined from tumor and control tissues. For high-quality RNA isolation, TRI Reagent^®^ (Sigma-Aldrich, Darmstadt, Germany; cat. no. T9424-100 ML). was used. It is a mixture of phenol, guanidinium isothiocyanate, and other compounds that aim to lyse cells and inactivate endogenous RNAses. 50 mg of the material collected from patients was homogenised with TRI Reagent and incubated for 5 min at 25°C. To separate the mixture of RNA, DNA, and protein, 0.2 mL of chloroform was added. The resulting mixture was incubated for 3 min at 25°C, and then the phases were separated by centrifugation in a microcentrifuge at 12,000× *g* for 15 min at 4°C. The upper phase, where the RNA accumulated, was isolated and treated with 0.5 mL of isopropanol to precipitate. This mixture was incubated for 10 min at 25°C, then centrifuged again in a microcentrifuge at a speed of 12,000× *g* for 10 min at a temperature of 4°C. The supernatant thus formed was removed. The remaining RNA pellet, after washing with 1 mL of 75% ethanol, was centrifuged in a microcentrifuge at 7500× *g* for 5 min at 4°C. The resulting precipitate was dried and then dissolved in 30 μL of ultrapure water. The mixture was then incubated at 55°C for 10 min. The purity and concentration of the RNA were determined using a NanoDrop Maestro Nano spectrophotometer (Maestrogen, Hsinchu, Taiwan). For subsequent analysis, the high-purity RNA exhibited an A260/280 ratio within the range of 1.8 to 2.0. The isolated mRNA was frozen at −80°C before further examination.

#### 2.3.2 Assessment of MYO18A Expression

To obtain cDNA using the isolated mRNA, reverse transcription was performed using the NG dART RT-PCR (Next Generation—Diversity Arrays Technology Real Time Polymerase Chain Reaction) kit (EURx Ltd., Gdańsk, Poland; cat. no. E0802-02) according to the recommendations provided by the manufacturer. Firstly, a reaction mixture was prepared containing 10 µL of isolated RNA (200 ng/µL), 2 µL of 10× RT Buffer, 0.8 µL of 25× dNTP Mix (100 mM), 2 µL of 10× RT random primers, 1 µL of MultiScribe reverse transcriptase (50 U/µL), 0.5 µL of RNAse inhibitor (40 U/µL) and 3.2 µL of RNAse-free water, in triplicates. The reaction was performed using a Mastercycler gradient thermal cycler (Eppendorf, Germany) for 10 min at 25°C, then for 50 min at 50°C, and finally for 5 min at 85°C.

The relative expression of the tested *MYO18A* (Hs00373018_m1, Thermofisher, Waltham, MA, USA) was measured by the ΔΔCt method (comparative threshold cycle method) using the ACTB and RNA18S5 genes (Hs01060665_g1, Hs03928990_g1, Thermofisher) [26–30]. The reference genes were selected based on preliminary analysis, which aimed at determining variability under experimental conditions, and according to the requirements of MIQE Guidelines [31]. To assess the consistency between the two reference genes, we calculated the Pearson correlation coefficient (r) between the raw Cq values of ACTB and RNA18S5 across all samples. The correlation was high (r = 0.878), indicating that both genes exhibited similar expression trends throughout the dataset. According to BestKeeper criteria, such a strong correlation supports the use of a combined normalization factor. Therefore, normalization was performed using the geometric mean of ACTB and RNA18S5, which provides greater stability than either gene alone and reduces sample-specific variability [31].

For endogenous qPCR control, the Real-time PCR 7500 fast system (Applied Biosystems, USA) and Fast Probe qPCR Master Mix (2×) reagents (EURx, Poland) were used in accordance with the manufacturers’ instructions. Briefly, a reaction mixture containing 1 µL of the cDNA (5 ng), 10 µL of Fast Probe qPCR Master Mix (2×), 9 µL of RNase–free water, 0.5 µL of ROX (carboxy-X-rhodamine) Solution (50 nM), and 0.5 µM of gene–specific TaqMan probe was prepared. Thermal profile of the reactions performed: 20 s at 95°C, followed by 40 cycles of 3 s at 95°C and 30 s at 60°C. The reactions were carried out in three technical replicates. Data are presented as fold changes in RQ (relative quantification) mRNA levels.

The following formulas were used:

ΔCt (unknown sample) = Ct of the test gene − Ct of the reference gene

ΔCt (calibrator) = Ct of the test gene − Ct of the reference gene

ΔΔCt = ΔCt (unknown sample) − ΔCt (calibrator)

(RQ = 2^−ΔΔCt^)

Legend: Δ—delta; Ct—cycle threshold

### 2.4 Statistical Evaluation of Research Results

Statistical studies were performed in R (version 4.2.2), a free programming language used for statistical analyzes (https://cran.r-project.org) on the RStudio platform. The following libraries were used for the research: “lubridate” for data operations, “readxl” for loading data from the database, “asbio” for contrast studies, “corplot” and “spearmanCI” for correlation analysis, “survival” and “survminer” for survival analysis, and “ggplot2” for plotting.

A 95% confidence interval (CI) was assumed. The level of significance was set at 0.05 (*p* ≤ 0.05). This means that the verified null hypothesis should be rejected when the probability of its truth does not exceed 5%. If the probability of the hypothesis being true is greater than 5%, there are no grounds to reject it.

Before starting statistical analyses, it was checked whether the *MYO18A* feature was normally distributed in order to apply appropriate tests for further analyses. Therefore, we compute for the *MYO18A* the following tests for normality: Cramer-von Mises, Anderson-Darling, Lilliefors (Kolmogorov-Smirnov), Jarque Bera, Shapiro-Wilk, Shapiro-Francia, and Pearson chi-square normality test, obtaining *p* between 0 and 7.37 × 10^−10^, thus the MYO18A isn’t normally distributed. In consequence, we use later nonparametric tests for MYO18A, such as the Kruskal–Wallis rank sum (nonparametric ANOVA) test for greater than two groups or test Mann Whitney U (Wilcoxon) for comparison of two groups.

The next analysis concerned the examination of the significance of differences between three groups—two groups of gliomas: grade G3 and G4, and grade G1 and G2, and the third group was the control group. The null hypothesis was: “There are no differences in the *MYO18A* RQ value between the two glioma groups and the control group.”

Since *p* = 0.0021 < 0.05, the null hypothesis was rejected, the alternative hypothesis was accepted, which means: “There are differences in *MYO18A RQ* values between the G3/G4, G1/G2 and normal glioma groups” (Kruskal-Wallis test). For the post-hoc comparison, we computed the U Mann-Whitney test with correction of Bonferroni.

Then, for each of the nominal features (bivalent and multivalued) and ordinal, RQ values for MYO18A were examined in those determined by these characteristic groups using non-parametric tests.

Spearman correlation was calculated between quantitative features to assess how strongly two sets of ranks are correlated, i.e., how well the relationship between two variables can be described using a monotonic function (it differs from Pearson correlation, which assesses linear relationships only). Test for significance (hypothesis *ρ* = 0) using

$$\rho \times \frac{\sqrt{n-2}}{\sqrt{1-\rho^{2}}}$$

where *n* is the number of observations *and ρ* is the Spearman correlation coefficient), which is distributed approximately as Student’s *t*-distribution with n − 2 degrees of freedom under the null hypothesis. Additionally, the 0.95% confidence interval of Spearman’s *ρ* can be easily obtained using the Jackknife Euclidean likelihood approach in de Carvalho and Marques [32] and implemented in the R library spearmanCI.

For survival analysis, the Kaplan-Meyer estimators for the evaluation of survival probabilities and PFS probabilities were constructed for the different groups divided according to the histological grade of the tumor, age (cut at the median 52), and other nominal characteristics. The survivals of different groups were compared by the $\chi^{2}$ (chi squared) test.

The MYO18A RQ values were compared within groups of patients with the same grade of malignancy using the U Mann-Whitney test. Each group: G2, G3, and G4 was divided according to increasing PFS and survival time. The *MYO18A* values of people living shorter than the group average were then compared with those of people living longer than the group average.

## 3 Research Results

### 3.1 Characteristics of Patients Depending on Histopathological Diagnosis

The mean age was 51.9 years (SD—standard deviation, ±13.4). For patients diagnosed with grade 4 glioma, the average age was 58.4 years, for grade 3–46.6 years, for grade 2–36.8 years, and for grade 1–44 years. Patient characteristics are presented in Table 1.

**Table 1:** Patient and tumor characteristics.

PP	Age, years	Sex	Histopathological diagnosis	Tumor size (mm) and Tumor volume (V, mL)	Tumor location
**Glioma grade 4**					
1	65	M	Glioblastoma multiforme	40 × 70 × 40 (mm) V = 58.2 mL	Left parietal lobe
2	37	M	Astrocytoma	55 × 65 × 50 (mm) V = 94.7 mL	Left temporal and parietal lobe
3	65	M	Glioblastoma multiforme	40 × 45 × 50 (mm) V = 47.7 mL	Right parietal and occipital lobe
4	46	M	Glioblastoma multiforme	32 × 31 × 30 (mm) V = 15.8 mL	Left frontal lobe
5	69	M	Glioblastoma multiforme	70 × 80 × 70 (mm) V = 207.8 mL	Right temporal lobe
6	54	M	Glioblastoma multiforme	38 × 54 × 40 (mm) V = 43.5 mL	Right temporal lobe
7	56	F	Glioblastoma multiforme	38 × 31 × 35 (mm) V = 21.9 mL	Right temporal lobe
8	67	M	Glioblastoma multiforme	52 × 47 × 34 (mm) V = 44.0 mL	Right parieto-occipital lobe
9	43	M	Glioblastoma multiforme	36 × 21 × 12 (mm) V = 4.9 mL	Left frontal lobe
10	43	M	Glioblastoma multiforme	26 × 31 × 18 (mm) V = 7.7 mL	Left temporal and parietal lobe
11	62	M	Glioblastoma multiforme	80 × 58 × 50 (mm) V = 123.0 mL	Left temporal and parietal lobe
12	48	F	Glioblastoma multiforme	70 × 45 × 50 (mm) V = 83.5 mL	Left frontal lobe
13	71	M	Glioblastoma multiforme	58 × 39 × 40 (mm) V = 48.0 mL	Left temporal lobe
14	52	M	Glioblastoma multiforme	44 × 62 × 39 (mm) V = 56.4 mL	Right occipital lobe
15	51	M	Glioblastoma multiforme	45 × 30 × 33 (mm) V = 23.6 mL	Left temporal lobe
16	49	M	Astrocytoma	50 × 60 × 40 (mm) V = 63.6 mL	Right frontal lobe
17	52	F	Glioblastoma multiforme	36 × 47 × 46 (mm) V = 41.2 mL	Left temporal and parietal lobe
18	69	F	Glioblastoma multiforme	60 × 45 × 50 (mm) V = 71.6 mL	Left temporal lobe
19	68	M	Glioblastoma multiforme	43 × 55 × 45 (mm) V = 56.4 mL	Left temporal lobe
20	63	M	Glioblastoma multiforme	26 × 22 × 20 (mm) V = 6.0 mL	Left parietal lobe
21	80	M	Glioblastoma multiforme	30 × 33 × 26 (mm) V = 13.6 mL	Left temporal lobe
22	66	F	Glioblastoma multiforme	40 × 35 × 50 (mm) V = 37.1 mL	Right temporal lobe
23	52	M	Glioblastoma multiforme	44 × 52 × 47 (mm) V = 57.0 mL	Left temporal lobe
24	66	F	Astrocytoma	30 × 40 × 35 (mm) V = 22.3 mL	Left parietal lobe
25	71	F	Glioblastoma multiforme	49 × 60 × 45 (mm) V = 70.1 mL	Right occipital lobe
26	51	M	Glioblastoma multiforme	62 × 46 × 57 (mm) V = 86.2 mL	Right frontal and parietal lobe
27	63	M	Glioblastoma multiforme	80 × 48 × 50 (mm) V = 101.8 mL	Left temporal lobe
***x*^–^ = 58.4**			***x*^–^V = 55.9 mL**		
**SD = 10.5**			**SD = 42.4 mL**		
**Glioma grade 3**					
28	39	M	Astrocytoma	78 × 32 × 31 (mm) V = 41.0 mL	Right frontal and parietal lobe
29	72	M	Astrocytoma	50 × 75 × 49 (mm) V = 97.4 mL	Left temporal lobe
30	56	M	Astrocytoma	60 × 55 × 59 (mm) V = 103.2 mL	Right parietal lobe
31	34	M	Astrocytoma	58 × 56 × 61 (mm) V = 105.0 mL	Right frontal lobe
32	48	F	Astrocytoma IDH	52 × 51 × 54 (mm) V = 75.9 mL	Right parietal and occipital lobe
33	55	F	Oligodendroglioma	62 × 64 × 54 (mm) V = 113.6 mL	Right frontal and parietal lobe
34	39	M	Astrocytoma IDH	55 × 65 × 50 (mm) V = 94.7 mL	Right temporal lobe
35	40	F	High-grade astrocytoma with piloid features	53 × 69 × 52 (mm) V = 100.8 mL	Right temporal lobe
36	37	M	Oligodendroglioma	68 × 38 × 55 (mm) V = 77.3 mL	Right frontal and temporal lobe
***x*^–^ = 46.7**			***x*^–^ = 90.0 mL**		
**SD = 11.6**			**SD = 20.8 mL**		
**Glioma grade 2**					
37	29	M	Oligodendroglioma	20 × 28 × 15 (mm) V = 4.4 mL	Right frontal lobe
38	41	F	Oligodendroglioma	20 × 10 × 15 (mm) V = 1.6 mL	Right frontal lobe
39	44	F	Oligodendroglioma	50 × 60 × 40 (mm) V = 63.6 mL	Right frontal lobe
40	32	F	Astrocytoma NEC, IDH-wildtype	25 × 32 × 23 (mm) V = 9.8 mL	Left temporal lobe
41	52	M	Astrocytoma	60 × 59 × 70 (mm) V = 131.3 mL	Right frontal lobe
42	22	M	Astrocytoma NEC, IDH-wildtype	32 × 37 × 23 (mm) V = 14.4 mL	Right temporal lobe
43	32	F	Astrocytoma	35 × 22 × 28 (mm) V = 15.5 mL	Left frontal and occipital lobe
44	43	M	Astrocytoma	43 × 39 × 36 (mm) V = 32.0 mL	Right temporal lobe
***x*^–^ = 36.9**			***x*^–^V = 34.1 mL**		
**SD = 9.1**			**SD = 41.2 mL**		
**Glioma grade 1**					
45	44	F	Pilocytic astrocytoma	24 × 13 × 16 (mm) V = 2.6 mL	Left temporal lobe
***x*^–^ = 44**					

Note: Abb: *x*^−^—arithmetic mean; SD—standard deviation; F—woman; M—man; NEC—Not Elsewhere Classified; V—tumor volume.

### 3.2 Assessment of Survival and PFS Depending on the Histopathological Diagnosis

PFS and OS for individual patients are presented in Table 2.

**Table 2:** *MYO18A* values expressed as RQ value, PFS time, and OS time in months—division depending on histopathological diagnosis.

Pr	Age, Years	Sex	Histopathological Diagnosis	*MYO18A* RQ Value	PFS, Months	OS, Months
**Glioma grade 4**						
1	65	M	Glioblastoma Multiforme	0.887	7.9	15.2
2	37	M	Astrocytoma	3.049	5.6	9.3
3	65	M	Glioblastoma Multiforme	0.308	2.0	8.5
4	46	M	Glioblastoma Multiforme	0.449	29.3	59.2
5	69	M	Glioblastoma Multiforme	0.934	6.1	8.0
6	54	M	Glioblastoma Multiforme	0.539	9.4	12.3
7	56	F	Glioblastoma Multiforme	0.35	12.6	15.0
8	67	M	Glioblastoma Multiforme	0.594	3.5	13.7
9	43	M	Glioblastoma Multiforme	7.157	5.97	22.2
10	43	M	Glioblastoma Multiforme	0.559	12.9	16.3
11	62	M	Glioblastoma Multiforme	0.406	0.8	0.8
12	48	F	Glioblastoma Multiforme	1.176	3.6	4.0
13	71	M	Glioblastoma Multiforme	0.34	1.8	7.2
14	52	M	Glioblastoma Multiforme	0.273	17.0	22.6
15	51	M	Glioblastoma Multiforme	0.329	28.3	36.4
16	49	M	Astrocytoma	0.314	3.0	5.3
17	52	F	Glioblastoma Multiforme	0.173	13.2	16.9
18	69	F	Glioblastoma Multiforme	1.104	6.7	10.8
19	68	M	Glioblastoma Multiforme	0.707	5.5	28.8
20	63	M	Glioblastoma Multiforme	0.435	6.3	11.3
21	80	M	Glioblastoma Multiforme	0.021	10.0	16.2
22	66	F	Glioblastoma Multiforme	0.138	9.8	14.2
23	52	M	Glioblastoma Multiforme	0.225	6.2	9.8
24	66	F	Astrocytoma	0.49	5.1	5.2
25	71	F	Glioblastoma Multiforme	0.178	6.9	9.4
26	51	M	Glioblastoma Multiforme	0.632	6.1	26
27	63	M	Glioblastoma Multiforme	0.426	0.6	0.6
				***x*^–^ = 0.822**	***x*^–^ = 8.4**	***x*^–^ = 15.0**
				**SD = 1.362**	**SD = 6.9**	**SD = 11.9**
**Glioma grade 3**						
28	39	M	Astrocytoma	0.437	8.7	10.5
29	72	M	Astrocytoma	0.171	6.6	13.2
30	56	M	Astrocytoma	0.432	12.5	22.5
31	34	M	Astrocytoma	1.296	21.6	21.7
32	48	F	Astrocytoma	1.163	N/A	72.5
33	55	F	Oligodendroglioma	0.102	10.7	17.2
34	39	M	Astrocytoma	0.414	9.8	14.4
35	40	F	High-grade astrocytoma with piloid features	0.284	12.4	20.4
36	37	M	Oligodendroglioma	0.215	13.8	20.9
				***x*^–^ = 0.501**	***x*^–^ = 18.7**	***x*^–^ = 23.7**
				**SD = 0.406**	**SD = 19.4**	**SD = 16.7**
				***x*^–^ = 0.742 (for G3 and G4)**	***x*^–^ = 10.9 (for G3 and G4)**	***x*^–^ = 17.2 (for G3 and G4)**
				**SD = 1.205 (for G3 and G4)**	**SD = 12.3 (for G3 and G4)**	**SD = 14.1 (for G3 and G4)**
**Glioma grade 2**						
37	29	M	Oligodendroglioma	0.93	N/A	59.2
38	41	F	Oligodendroglioma	0.13	47.9	116.7
39	44	F	Oligodendroglioma	0.133	N/A	70.2
40	32	F	Astrocytoma NEC, IDH-wildtype	1.041	29.7	42.2
41	52	M	Astrocytoma	0.815	56.3	60.9
42	22	M	Astrocytoma NEC, IDH-wildtype	0.272	38.9	72.7
43	32	F	Astrocytoma	0.19	50.2	70.8
44	43	M	Astrocytoma	0.014	N/A	46.7
				***x*^–^ = 0.440**	***x*^–^ = 49.9**	***x*^–^ = 67.4**
				**SD = 0.388**	**SD = 11.6**	**SD = 21.3**
**Glioma grade 1**						
45	44	F	Pilocytic astrocytoma	0.455	N/A	78.0
				***x*^–^ = 0.455**	**N/A**	***x*^–^ = 78.0**
				***x*^–^ = 0.442 (for G1 and G2)**	***x*^–^ = 53.0 (for G1 and G2)**	***x*^–^ = 68.6 (for G1 and G2)**
				**SD = 0.365 (for G1 and G2)**	**SD = 14.1 (for G1 and G2)**	**SD = 20.4 (for G1 and G2)**
**Control group**				0.891	N/A	N/A
				0.865	N/A	N/A
				1.096	N/A	N/A
				0.899	N/A	N/A
				1.148	N/A	N/A
				1.214	N/A	N/A
				1.179	N/A	N/A
				1.215	N/A	N/A
				0.758	N/A	N/A
				0.87	N/A	N/A
				***x*^–^ = 1.013**	N/A	N/A
				**SD = 0.164**	N/A	N/A

Note: Abb: PFS—progression-free survival; OS—overall survival; *x*^–^—arithmetic mean; SD—standard deviation; NEC—Not Elsewhere Classified; N/A—not applicable. For patients who did not have disease progression, PFS was taken as the OS.

### 3.3 Survival and PFS Analysis

**Figure 2:**
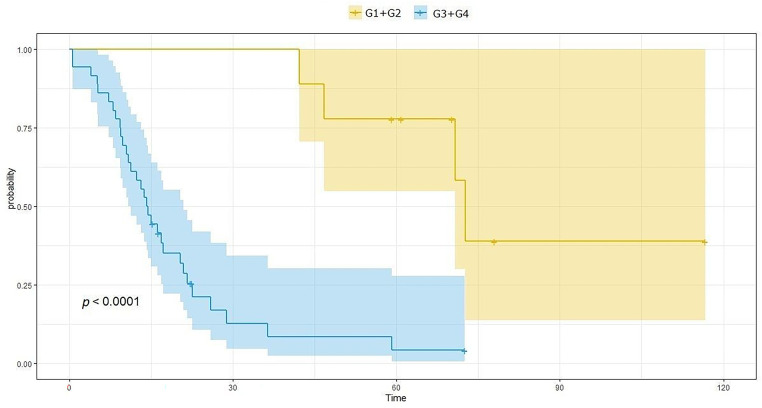
Overall survival (OS) length depends on the histological grade of the tumor. The image shows the Kaplan-Meier curve of the probability of OS as a function of time. The blue curve represents patients with malignancy grades G3 and G4, while the yellow line represents patients with malignancy grades G1 and G2.

**Figure 3:**
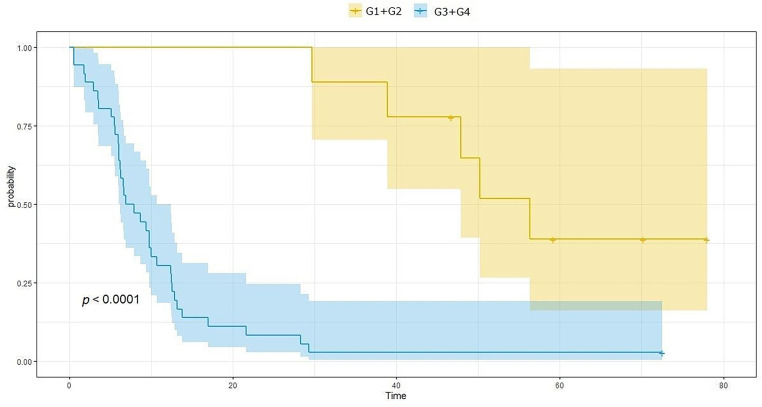
Progression-free survival (PFS) length depends on the histological grade of the tumor. The figure shows the Kaplan-Meier curve of PFS probability as a function of time. The blue curve represents patients with G3 and G4 malignancy, while the yellow line represents patients with G1 and G2 malignancy.

According to the analysis, the average survival time in patients treated due to brain gliomas was 27.46 months (SD = 25.8 months), and the average time to recurrence was 19.37 months (SD = 21.05 months). OS in the G3/G4 group of patients is significantly shorter than in the G1/G2 group and amounts to 17.2 months (SD = 14.1) and 68.6 months (SD = 20.4), respectively, *p* < 0.0001. Also, PFS in the G3/G4 group of patients is significantly shorter than in the G1/G2 group and amounts to 10.9 months (SD = 12.3) and 53 months (SD = 14.1), respectively, *p* < 0.0001. Figs. 2 and 3 show OS and PFS depending on the histological grade of the tumor.

**Figure 4:**
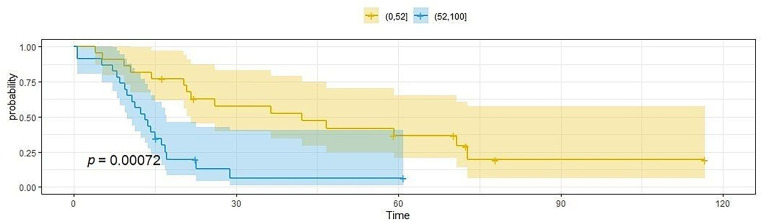
The length of OS depends on the patient’s age; the cut-off point was 52 years of age. The image shows the Kaplan-Meier curve of the probability of OS as a function of time. The blue curve represents patients aged up to 52 years, while the yellow curve represents patients over 52 years of age.

**Figure 5:**
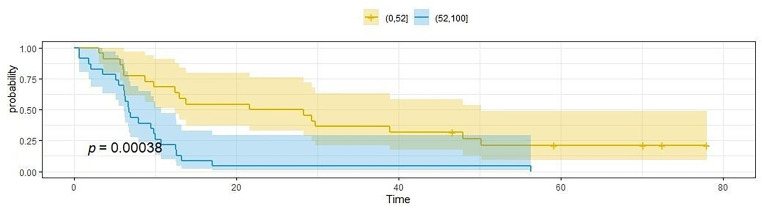
The length of PFS depends on the patient’s age, the cut-off point was 52 years of age. The figure shows the Kaplan-Meier curve of PFS probability as a function of time. The blue curve represents patients aged up to 52 years, while the yellow curve represents patients over 52 years of age.

Another analysis showed the impact of age on OS and PFS. The median age of patients was 52 years. Medium OS and the average PFS in the group of patients over 52 years of age were significantly shorter than in patients below 52 years of age. The mean OS in the first group was 12.1 months (SD = 6.6), in the second group 38.7 months (SD = 28.7), *p* < 0.0007. Similarly, the average PFS in the first group was 6,6 months (SD = 3.6), in the second group it was 28.7 months (SD = 23.4), *p* < 0.0004. Figs. 4 and 5 show survival (OS) and PFS depending on the age of the patients

### 3.4 MYO18A Expression Results

The results of MYO18A RQ expression tests are presented in Table 2. The Kruskal-Wallis test for MYO18A RQ values showed significant statistical differences in MYO18A RQ activity between groups of patients treated for G1/G2 gliomas and patients treated for G3/G4 gliomas and the control group ($\chi^{2}$ = 12.362; df = 2; *p* = 0.0021). Contrast analysis for MYO18A RQ showed differences between control and G1/G2 group (W = 79, *p* = 0.0041) and between control and G3/G4 group (W = 302, *p* = 0.0012) and no significant differences between G1/G2 and G3/G4 groups (W = 127, *p* = 0.3276).

Analysis with G1 rejection was also performed—no statistical differences were found between groups of patients with glioma (G2, G3, G4) in MYO18A expression (Supplementary materials—Figs. S1 and S2).

The U Mann-Whitney test showed higher MYO18A values in patients diagnosed with grade G4 glioma among those with a shorter PFS time and those living shorter than the group average. However, statistically significant differences were achieved only for PFS for the MYO18A RQ feature (PFS = 4.64, SD = 2.16 vs. PFS = 15.83 and SD = 7.27, *p* = 0.0231).

### 3.5 Multivariate Correlation Results

**Figure 6:**
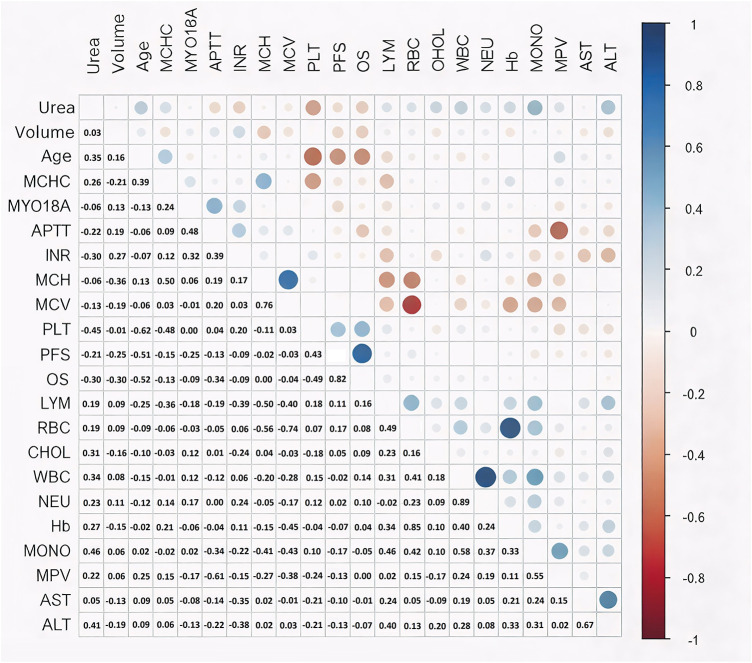
Spearman correlation. Blue color—positive correlation, red color—negative correlation. The strength of the correlation increases with color saturation and circle size. Correlation: weak 0.1–0.29; medium 0.3–0.5; hight >0.5. Abb: Urea—urea level; Volume—tumor volume; Age—patient’s age; MCHC—mean corpuscularhemoglobin concentration; MYO18A—Myosin 18A; APTT—activated partial thromboplastin time; INR—international normalised ratio; MCH—mean cell hemoglobin; MCV—mean cell volume; PLT—platelets; LYM—lymphocytes; RBC—red blood cells; CHOL—cholesterol level; WBC—white blood cells; NEU—neutrohils; Hb—hemoglobin; MONO—monocytes; MPV—mean platelet volume; AST—aspartate aminotransferase; ALT—alanine transaminase. A medium positive correlation was found between MYO18A RQ and tumour volume, APTT and INR.

The occurrence of correlation between MYO18A values and morphological and biochemical blood parameters was also tested, as well as the correlation between MYO18A values and OS and PFS. Fig. 6 shows Spearman correlation values.

Blood test results, PFS, OS, and MYO18A values are listed in the supplementary materials (Table S1).

The highest and statistically significant positive correlation in the case of MYO18A RQ was demonstrated with APTT (0.48, CI = [0.2180; 0.6785], *p* = 0.0008) and INR (0.32, CI = [0.0321; 0.5628], *p* = 0.0307).

There was also a weak positive correlation with the number of neutrophils (0.17, CI = [−0.1324; 0.4396], *p* = 0.2709), tumor volume (0.13, CI = [−0.1711; 0.4070], *p* = 0.3989), cholesterol level (0.12, CI = [−0.1766; 0.4023], *p* = 0.4194) and white blood cell count (0.12, CI = [−0.1763; 0.4026], *p* = 0.4183) and a negative correlation with PFS (−0.25, CI = [−0.5181; 0.0705], *p* = 0.1254), and a weak negative correlation with the number of lymphocytes (−0.18, CI = [−0.4453; 0.1154], *p* = 0.2254), the MPV value (−0.17, CI = [−0.4410; 0.1306], *p* = 0.2658). ALT (−0.13, CI = [−0.4067; 0.1715], *p* = 0.4004), and the age of the patient (−0.13, CI = [−0.4042; 0.1743], *p* = 0.4108), although not significant, too.

## 4 Discussion

Brain tumors have a poor prognosis. Over the last three decades, their incidence has almost doubled. In Poland, approximately 1.5% of the total cancer incidence is brain tumors. Women are affected slightly less frequently than men—1.4% and 1.6% of new cases, while the mortality rate is 3% and 2.7%, respectively [3,33].

In Poland, brain tumors do not rank among the top ten cancer cases, but in terms of mortality, they already rank 10th, which proves the diagnostic and therapeutic problem these tumors cause in oncology. The results of treatment are primarily determined by the possibility of performing radical surgery, but it should be noted that the radical nature of surgery is limited due to the possibility of complications related to brain damage. That is why postoperative management of gliomas, especially grades G3 and G4, is so important. Currently, the treatment of choice is the addition of radiochemotherapy to surgical treatment. This approach led to improved treatment outcomes. In the studied group of G4 patients, the average OS was 15 months, which is consistent with the average results in the literature (15.6 months) [3,34].

Also, in the case of the remaining degrees of differentiation, the treatment results achieved were similar to literature data. The influence of age on the prognosis of patients has also been demonstrated. Younger patients had a better prognosis than older patients over 52 years of age. Patients over 52 years of age had shorter PFS and OS, which is also associated with a lower degree of tumor differentiation, occurring more often in older patients. The average age of patients in this study treated for G4 tumors was 58.4 years, G3—46.7 years, G2—36.9 years, and G1—44 years. The obtained results are consistent with the characteristics of the incidence of brain gliomas [4]. This proves that the studied group is representative of brain gliomas.

Brain gliomas belong to a group of tumors with low sensitivity to radiotherapy or chemotherapy, which is why it is so important to understand the changes leading to neoplastic transformation in normal glial cells, which may enable the search for new treatment methods [34].

MYO18A, which was detected in 2000 by Obinata et al., is a protein belonging to the myosin family that binds F-actin [35]. As a result of research conducted in recent years, *MYO18A* has been classified as one of the genes playing an important role in the process of carcinogenesis [36]. This is due to both the role of the Golgi apparatus in the cell and its participation in the processes of post-translational protein modification and lipid modification, as well as the participation of this gene in cellular pathways, including important pathways responsible for cell proliferation, division, and apoptosis.

*MYO18A* is expressed in all human tissues, including the brain. It has not been shown to be specific to any particular brain region [37]. MYO18A has recently been classified as an oncoprotein [36,38], and its increased level is expected to result in increased invasiveness of cancer cells. Nevertheless, the results of cancer studies are not clear [36,38].

Due to the lack of key amino acid residues, MYO18A is an inactive motor domain, unlike other myosin isoforms. The MYO18B isoform, which is found primarily in skeletal muscle, is also expected to lack this domain. MYO18A, despite the presence of a long spiral, cannot form fibers, but it copolymerizes with NM2A (*non-muscle myosin 2A*), which leads to a reduction in the number of NM2A molecules and thus the length of these fibers [39].

In the case of the PC-3 prostate cancer cell line, it was shown that overexpression of *MYO18A* brings NM2A fibers closer to the plasma membrane, which is believed to promote the formation of metastases [40]. The role of *MYO18A* in the cancer process may be indicated by the presence of a fusion of *MYO18A* with *PDGFRB* (*platelet-derived growth factor receptor beta*) in the myeloproliferative syndrome [41].

The N-terminal part of the MYO18A protein has a repeated Lys-Glu (KE) region (lysine-glutamic acid) and a small protein module—the PDZ domain, composed of about 90 amino acids, characteristic for each species. The PDZ domain was initially found in the following proteins: PSD-95 (*post-synaptic density*, occurs in synaptic connections), DlgA (*protein disc large*, precursor of the family of guanine kinases associated with membranes in Drosophila) and ZO-1 (*protein zonula occludens-1*, an actin filament-binding component of intercellular junctions). It has the ability to form protein-protein and protein-lipid bonds. The N-terminal PDZ domain of MYO18A binds to membrane proteins that can localize NM2A fibers in the plasma membrane [42]. Possession of the PDZ domain would indicate the involvement of MYO18A in signaling pathways. This thesis is supported by tyrosine phosphorylation in MYO18A myeloblastic cells after stimulation with macrophage colony-stimulating factor (CSF-1) [41]. Other proteomic analyzes also showed phosphorylation of MYO18A on serine, threonine, and tyrosine (Ser, Thr, and Tyr) and other post-translational modifications (lysine ubiquitination, methylation, acetylation) [43]. MYO18A is expected to participate by forming the MRCK-LURAP1-MYO18A complex (MRCK—*myotonic dystrophy kinase*; LURAP1—*leucine-rich adapter protein 1*) [44] in the retrograde flow in the actinomyosin network in cell laminae and in the cell. As further research showed, this complex is responsible for proper cell migration. In the analysis of the breast cancer database from the Cancer Genome Atlas, Sanchez-Garcia et al. [45] identified new oncogenes, including *MYO18A*, in addition to known oncogenes such as *MYC* and *HER2/Neu*. However, there is no experimental evidence to confirm the involvement of this gene in the process of carcinogenesis [46].

It is overexpressed in metastatic prostate cancer cell lines (PC-3) [40], but has not been demonstrated in prostate cancer cells taken from the tumor. Overexpression of *MYO18A* contributes to the reduction of NM2A stress fibers and the predominant localization of NM2 near the plasma membrane. In cancer, *MYO18A* is also expected to fuse with *PDGFR* (*platelet-derived growth factor receptor*), *FGFR* (*fibroblast growth factor receptor*), and MLL (*Mixed Lineage Leukemia*), which indicates the involvement of *MYO18A* in the cancer process [41,47,48].

However, the association of this gene with cancer has not been proven, unlike the *MYO18B* isoform. *MYO18B* is considered a tumor suppressor gene, the lack of expression of which has been found in cell lines of lung cancer, pleural mesothelioma, breast cancer, ovarian cancer, and pancreatic cancer [21,49,50]. In clonogenic cells taken from a colorectal cancer tumor, it was shown that silencing *MYO18A* by introducing siRNA (*small interfering RNA*) complementary to *MYO18A* into the cell resulted in reduced migratory activity of clonogenic cells by 20% to 40% [36]. However, in cholangiocarcinoma, silencing of *MYO18A* was associated with greater proliferation, invasiveness, and migration. Although the level of *MYO18A* expression in tumor cells had no impact on the prognosis of patients with cholangiocarcinoma, such a correlation occurred in the case of additional overexpression of *SMAD4* (*Mothers against decapentaplegic homolog 4*) [38]. The SMAD4 protein is associated with the signaling pathway through the transforming growth factor β (TGFβ) pathway from the cytoplasm to the cell nucleus. Greater expression of both MYO18A and SMAD4 was associated with better patient prognosis than SMAD4 overexpression alone. This is most likely related to a decrease in the phosphorylation of p21-activated kinase (PAK1) at position 423 for threonine (PAK1-T423) and β-catenin-S675 (serine at position 675). MYO18A expression in CCA tissues was negatively associated with β-catenin-S675 and PAK1-T423 phosphorylation [38].

In a study on acute myeloid leukemia (AML), the levels of GOLPH3, MY018A, PITPNC1, and RAB1B proteins in blood serum were determined in patients. In the case of all tested proteins, their higher levels were found in the serum of AML patients compared to the control group of healthy people (*p* < 0.0010), as well as a worse prognosis in the case of higher levels of GOLPH3 (*p* = 0.014), MYO18A (*p* = 0.047), PITPNC1 (*p* = 0.008) and RAB1B (*p* = 0.033) [19].

In the work of Duhamel et al. [51], they demonstrated the importance of expression of proteins characteristic of the glial or progenitor cell lineage associated with neurodevelopmental genes, including *MYO18A*. To our knowledge, no data on the relationship between *MYO18A* expression and prognosis in brain gliomas are available in the literature. The study showed increased expression at the *MYO18A* mRNA level in G3/G4 gliomas (RQ = 0.742) compared to G1/G2 gliomas (RQ = 0.442). However, this difference was not statistically significant. There was a statistically significant difference in *MYO18A* expression between the control tissue and tumor cells at the G3/G4 and G1/G2 degrees of differentiation. Spearman’s correlation showed a negative correlation between *MYO18A* expression at the mRNA level and PFS (−0.25). However, in the case of OS, this value was −0.09, which indicates no or very low such correlation. A higher level of *MYO18A* expression probably affects the greater proliferative capacity of the tumor, as evidenced by the positive correlation shown in the Spearman correlation between tumor volume and *MYO18A* expression.

The U Mann-Whitney analysis confirmed the impact of *MYO18A* expression on PFS. It was shown that higher RQ values in patients treated for G4 brain glioma were associated with earlier recurrence, and this difference was statistically significant

MYO18A plays an important role in organising the cell cytoskeleton by interacting with NM2A (*non-muscle myosin II-A*) and F-actin [52]. F-actin is a crucial cytoskeletal protein that is responsible for cell migration and invasion in glioblastoma multiforme. *MYO18A* may also fuse with the *FGFR1* (*fibroblast growth factor receptor gene*), which is overexpressed in brain gliomas and associated with radiotherapy resistance [53]. The direct and indirect interactions of MYO18A with NM2A, LURAP1, and GOLPH3 may influence the cell cytoskeleton and related signalling pathways, thereby affecting tumour progression. NM2A blockade has been shown to inhibit glioblastoma proliferation and affect platelet size [23,54,55]. MPV has been shown to have prognostic significance in cancers such as lung, colon, and renal cancers [24,54]. In this study, however, only a weak negative correlation was found between MYO18A and MPV, while a relatively high positive correlation was observed with APTT and INR. Factor V and factor XIII of the coagulation system are transported and packaged into COP1 vesicles in the Golgi apparatus. MYO18A is a contractile protein that provides the tensile force necessary for forming channels and vesicles for extracellular transport. Its expression may influence the extracellular transport of proteins [54,55].

The formation of the GOLPH3-PI4P-MYO18A (*Golgi Phosphoprotein 3-phosphatidylinositol 4-phosphate-Myosin 18A*) complex is responsible for the association with F-actin, which leads to the formation of appropriate GA membrane structures, including the formation of transport vesicles. Reduced expression of GOLPH3 and MYO18A impairs the transport of substances from the GA to the cytoplasmic membrane [46,56]. While recent studies have demonstrated that membrane structure formation in GA requires the exclusive interaction of PI4P and GOLPH3, independent of MYO18A, a MYO18A deficiency is associated with impaired GA membrane curvature formation, GA tubule formation, and the transport of substances from GA to the cytoplasm or other cell structures. The GOLPH3-PI4P-MYO18A-F-actin complex influences the structure of the GA and plays a role in cell migration, thereby promoting the development of metastases in cancers [46,56,57].

Multivariate analysis also showed a negatively correlated OS (−0.34). APTT time, i.e., kaolin-cephalin time, tells us about the factors of the coagulation system, including plasma prekallikrein [58,59]. The activation of prekallikrein to kallikrein is by Factor XII. FA Kallikrein catalyzes the formation of bradykinin from its precursor, which increases epithelial permeability through B1 and B2 receptors present on endothelial cells. Stimulation of B2 receptors causes the release of prostaglandins and nitric oxide, thereby increasing vascular permeability. The increase in the expression of B1 receptors occurs under the influence of cytokines such as intreleukin 1β (IL-1β) or tumor necrosis factor α (TNFα) [58,59]. As a result of stimulation of these receptors by kinins, angiogenesis is stimulated, leading to tumor growth. The presence of these receptors has been demonstrated in brain glioma cells and vascular endothelium [59]. Pillat et al. [60] showed that stimulation of B1 and B2 receptors by bradykinin in the U-373 glioblastoma cell line causes an increase in the expression of these kinin receptors on glioma cells. An increase in the permeability of cerebral vessels under the influence of kinins may be one of the options for intensifying the treatment of brain tumors, where the permeability of cytostatics to the CNS is low, which is one of the reasons for the failure of chemical treatment of these tumors.

The above-mentioned studies indicate the complexity of carcinogenesis processes and the ambiguity of the role of individual genes, including Golgi apparatus protein genes. Many factors are involved in the process of carcinogenesis, and it is difficult to clearly demonstrate their predictive and prognostic importance. The interaction between them may determine whether a given factor becomes a factor promoting oncogenesis and, at the same time, may influence the course of the disease itself in the patient. Undoubtedly, Golgi proteins are among the proteins that can contribute to the understanding of oncogenesis and thus become themselves, or their genes, the target of new therapies, prompting further detailed research on this cellular organelle.

This study has certain limitations. One of them is the control tissue Control samples from patients who underwent surgery for tumors were not included. The small number of patients in the control group is also a weakness of this study. This is due to limited access to the study material. These limitations may have influenced the study results. Further studies with a larger group of patients are necessary.

## 5 Conclusions

Based on the conducted research, it seems that MYO18A can be considered a prognostic factor for PFS in patients treated for G4 gliomas, because higher MYO18A expression was associated with earlier recurrence in these patients. It also appears that MYO18A may have an impact on greater proliferative capacity. Research on MYO18A should be continued because the results are inconclusive, both in the study and in the available literature.

## Supplementary Materials







## Data Availability

The authors confirm that the data supporting the findings of this study are available within the article and its Supplementary Materials.

## Supplementary Materials

The supplementary material is available online at https://www.techscience.com/doi/10.32604/or.2026.074078/s1.
