# Supplementary figures and images for: MYO18A Expression is a Prognostic Factor for Progression-Free Survival in Grade 4 Adult gliomas. Preliminary Report
Source: Oncol Res. 2026 Apr 22;34(5):22. doi: 10.32604/or.2026.074078 (PMC13126409; doi:10.32604/or.2026.074078)

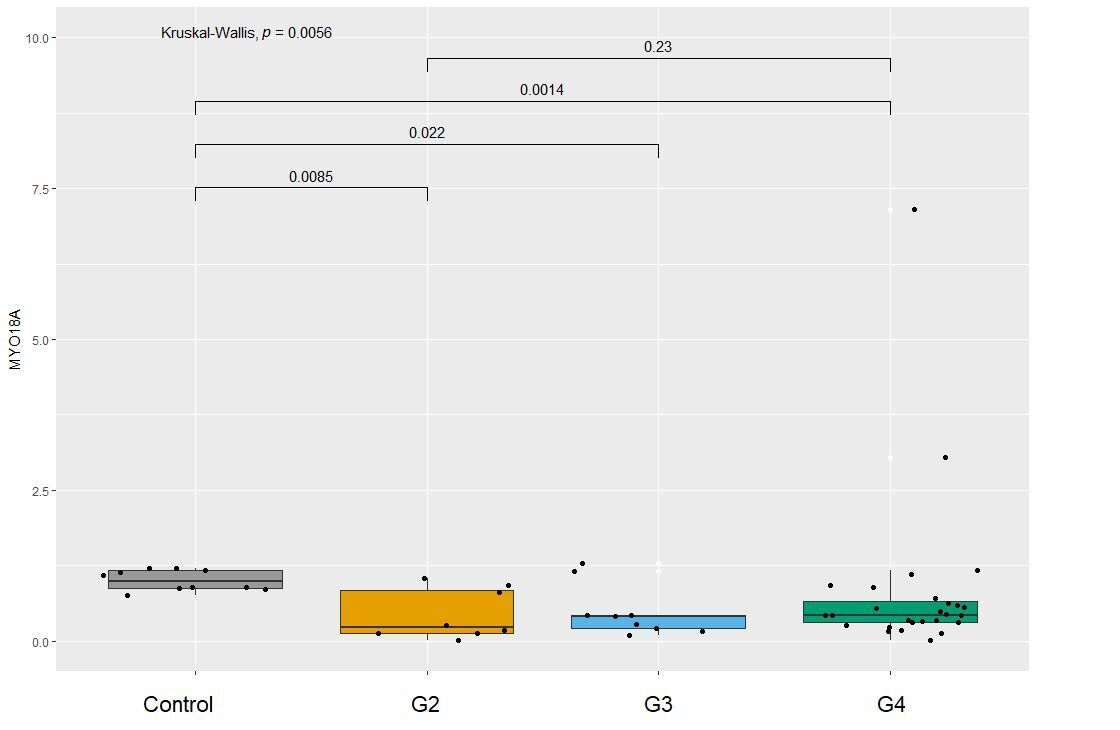

Supplement: Supplementary file 1 [file OncolRes-34-74078-s001.tif]

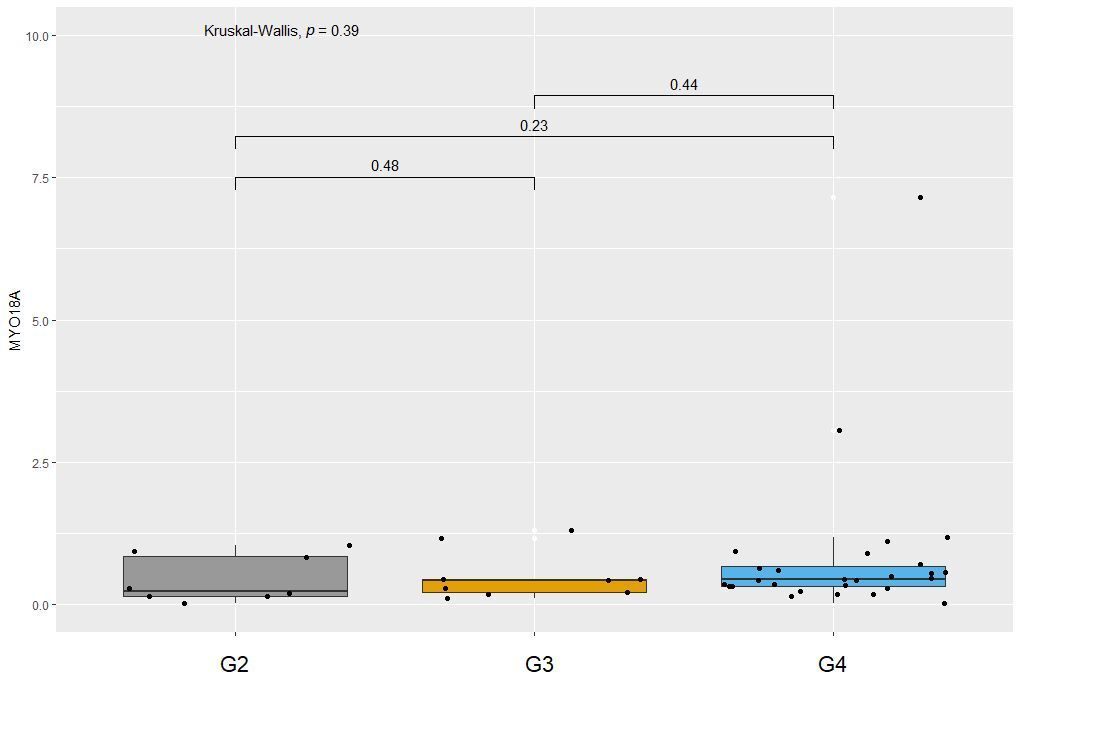

Supplement: Supplementary file 2 [file OncolRes-34-74078-s002.tif]
