# Supplementary material for: MYO18A Expression is a Prognostic Factor for Progression-Free Survival in Grade 4 Adult gliomas. Preliminary Report
Source: Oncol Res. 2026 Apr 22;34(5):22. doi: 10.32604/or.2026.074078 (PMC13126409; doi:10.32604/or.2026.074078)
Supplement: Supplementary file 3 [file OncolRes-34-74078-s003.docx]

**Suplementary materials:**

**Table S1. Blood test results, PFS, OS and MYO18A value**

| **Number** | **Hb** | **RBC** | **MCV** | **MCHC** | **MCH** | **PLT** | **MPV** | **WBC** | **LYM** | **NEU** | **MONO** | **APTT** | **INR** | **AST** | **ALT** | **CHOL** | **UREA** | **PFS** | **OS** | **MYO18A RQ** |
| --- | --- | --- | --- | --- | --- | --- | --- | --- | --- | --- | --- | --- | --- | --- | --- | --- | --- | --- | --- | --- |
| 1 | 13,6 | 4,41 | 90,9 | 34 | 30,9 | 225 | 6,6 | 11,85 | 1,35 | 9,35 | 0,74 | 31,2 | 1,02 | 27 | 52 | 219 | 47,9 | 7,9 | 15,2 | 0,887 |
| 2 | 16,1 | 5,94 | 79,7 | 34 | 27,1 | 362 | 7,5 | 9,41 | 1,94 | 6,31 | 0,86 | 31 | 1,2 | 24 | 36 | 141 | 25,7 | 5,6 | 9,3 | 3,049 |
| 3 | 15,5 | 5,4 | 87,1 | 32,9 | 28,7 | 165 | 10 | 8,8 | 3,07 | 4,32 | 1,03 | 25,8 | 1,01 | 27 | 22 | 159 | 37,3 | 2 | 8,5 | 0,308 |
| 4 | 16 | 5,75 | 82 | 33,9 | 27,8 | 165 | 8,8 | 8,21 | 2,01 | 5,42 | 0,54 | 25,7 | 0,95 | 18 | 36 | 247 | 48,8 | 29,3 | 59,2 | 0,449 |
| 5 | 12,6 | 3,67 | 98 | 35,4 | 34,4 | 158 | 8 | 7,22 | 1,16 | 5,36 | 0,48 | 32 | 0,99 | 21 | 21 | 213 | 59,4 | 6,1 | 8 | 0,934 |
| 6 | 14,2 | 4,68 | 94,9 | 32 | 30,3 | 262 | 7,6 | 5,38 | 1,07 | 3,65 | 0,47 | 27,1 | 0,98 | 22 | 24 | 200 | 16,2 | 9,4 | 12,3 | 0,539 |
| 7 | 11,9 | 3,7 | 92,6 | 34,7 | 32,1 | 162 | 7 | 6,77 | 1,06 | 5,36 | 0,23 | 27,4 | 1 | 14 | 22 | 218 | 34,2 | 12,6 | 15 | 0,35 |
| 8 | 15,1 | 4,98 | 88,4 | 34,3 | 30,3 | 218 | 7,1 | 10,03 | 2,15 | 6,87 | 0,65 | 31,7 | 1,05 | 49 | 48 | 239 | 27,5 | 3,5 | 13,7 | 0,594 |
| 9 | 12,6 | 3,87 | 95 | 34,3 | 32,5 | 209 | 7,1 | 6,15 | 0,95 | 4,67 | 0,3 | 29,3 | 1 | 16 | 14 | 223 | 16,5 | 5,9 | 22,2 | 7,157 |
| 10 | 12,6 | 3,87 | 95 | 34,3 | 32,5 | 209 | 7,1 | 6,15 | 0,95 | 4,67 | 0,3 | 29,3 | 1 | 16 | 14 | 223 | 16,5 | 12,9 | 16,3 | 0,559 |
| 11 | 12,8 | 4,32 | 89,8 | 32,9 | 27,6 | 143 | 7,8 | 7,34 | 1,51 | 5,17 | 0,52 | 25 | 0,86 | 24 | 41 | 201 | 48,9 | 0,6 | 0,6 | 0,426 |
| 12 | 14,2 | 4,82 | 82,4 | 32,3 | 29,5 | 218 | 8,8 | 14,65 | 1,49 | 12,12 | 0,76 | 28 | 1,1 | 12 | 10 | 244 | 42,5 | 3,6 | 4 | 1,176 |
| 13 | 13,6 | 4,44 | 89,9 | 34,1 | 30,6 | 251 | 10,3 | 4,52 | 0,81 | 2,83 | 0,76 | 21,5 | 1,08 | 14 | 27 | 218 | 50,8 | 1,8 | 7,2 | 0,34 |
| 14 | 18,3 | 5,74 | 92,2 | 34,6 | 31,9 | 215 | 6,8 | 12,89 | 1,72 | 10,49 | 0,5 | 24,7 | 1,08 | 13 | 26 | 235 | 69,8 | 17 | 22,6 | 0,273 |
| 15 | 15,7 | 5,25 | 88,3 | 33,8 | 29,8 | 187 | 6,9 | 5,21 | 1,32 | 3,13 | 0,37 | 28,9 | 1,1 | 18 | 19 | 236 | 38,1 | 28,3 | 36,4 | 0,329 |
| 16 | 13,7 | 4,03 | 100 | 33,9 | 33,9 | 194 | 7,1 | 5,75 | 2,04 | 3,12 | 0,33 | 28,9 | 1,13 | 17 | 35 | 223 | 27,1 | 3 | 5,3 | 0,314 |
| 17 | 14,4 | 5,41 | 82,4 | 32,2 | 26,5 | 217 | 6,9 | 5,41 | 1,89 | 2,97 | 0,34 | 26 | 0,96 | 20 | 27 | 209 | 28,4 | 13,2 | 16,9 | 0,173 |
| 18 | 13,2 | 3,44 | 108,4 | 35,5 | 38,5 | 192 | 7,1 | 4,48 | 0,85 | 2,92 | 0,5 | 28,7 | 1,1 | 16 | 18 | 150 | 31,7 | 6,7 | 10,8 | 1,104 |
| 19 | 14,3 | 4,63 | 86,6 | 35,6 | 30,8 | 129 | 10,5 | 9,97 | 0,77 | 8,63 | 0,44 | 22,9 | 1,2 | 30 | 37 | 181 | 29,7 | 5,5 | 28,8 | 0,707 |
| 20 | 13,8 | 4,03 | 97,5 | 34,8 | 36,7 | 151 | 8,9 | 7,23 | 0,82 | 5,87 | 0,45 | 25,6 | 1,1 | 24 | 31 | 166 | 30,8 | 6,3 | 11,3 | 0,435 |
| 21 | 13,9 | 4,34 | 93,71 | 34,5 | 33,5 | 172 | 8,6 | 6,26 | 1,02 | 5,04 | 0,42 | 26,4 | 0,91 | 22 | 29 | 176 | 30,1 | 10 | 16,2 | 0,021 |
| 22 | 14,8 | 4,79 | 88,7 | 34,8 | 30,9 | 141 | 11,5 | 11,51 | 1,22 | 9,5 | 0,71 | 21,6 | 0,95 | 47 | 29 | 247 | 38,4 | 9,8 | 14,2 | 0,138 |
| 23 | 14,2 | 4,25 | 94,8 | 35,1 | 34,2 | 158 | 9,2 | 7,75 | 0,88 | 6,4 | 0,47 | 25,4 | 1,05 | 29 | 27 | 185 | 33 | 6,2 | 9,8 | 0,225 |
| 24 | 13,5 | 4,7 | 86,3 | 33,9 | 28,5 | 153 | 8,6 | 7,28 | 1,5 | 5,11 | 0,62 | 24,9 | 0,94 | 20 | 28 | 225 | 40,5 | 5,1 | 5,2 | 0,49 |
| 25 | 13,5 | 4,6 | 85,7 | 34,2 | 28,9 | 156 | 8,9 | 7,32 | 1,62 | 5,13 | 0,6 | 25,1 | 0,96 | 19 | 29 | 227 | 38,9 | 6,9 | 9,4 | 0,178 |
| 26 | 14,3 | 4,91 | 83,9 | 34,7 | 29,1 | 164 | 9,3 | 7,21 | 1,42 | 5,1 | 0,68 | 24,9 | 1,04 | 14 | 16 | 251 | 29,3 | 6,1 | 26 | 0,632 |
| 27 | 12,8 | 4,32 | 89,8 | 32,9 | 27,9 | 143 | 7,8 | 7,34 | 1,51 | 5,17 | 0,52 | 25,3 | 0,86 | 24 | 41 | 201 | 48,9 | 0,6 | 0,6 | 0,426 |
| 28 | 15 | 4,7 | 95,7 | 33,4 | 32 | 218 | 6,5 | 9,98 | 0,95 | 8,35 | 0,43 | 29,7 | 1,1 | 19 | 48 | 277 | 36 | 8,7 | 10,5 | 0,437 |
| 29 | 14,6 | 4,9 | 85,9 | 34,6 | 29,7 | 195 | 7,8 | 4,97 | 1,26 | 3,2 | 0,36 | 27,2 | 1,02 | 19 | 16 | 170 | 28 | 6,6 | 13,2 | 0,171 |
| 30 | 15,9 | 5,39 | 82,6 | 35,7 | 29,5 | 256 | 9,8 | 12,49 | 1,57 | 9,59 | 1,3 | 24,1 | 1 | 14 | 23 | 180 | 52 | 12,5 | 22,5 | 0,432 |
| 31 | 13,4 | 4,6 | 89,9 | 33,4 | 30 | 226 | 6,7 | 3,62 | 1,46 | 1,65 | 0,29 | 29,6 | 1,05 | 24 | 20 | 205 | 24,3 | 21,6 | 21,7 | 1,296 |
| 32 | 12,6 | 4,34 | 94 | 31 | 29,2 | 323 | 8,2 | 11,49 | 1,19 | 9,75 | 0,43 | 26,4 | 1,13 | 13 | 12 | 204 | 26 | N/A | 72,5 | 1,163 |
| 33 | 13,2 | 4,51 | 92,1 | 32,1 | 29,7 | 274 | 7,6 | 7,55 | 1,48 | 5,6 | 0,45 | 28,2 | 1,09 | 18 | 16 | 196 | 25,3 | 10,7 | 17,2 | 0,102 |
| 34 | 13,7 | 4,68 | 88,9 | 33,1 | 28,5 | 268 | 8,2 | 8,62 | 1,35 | 6,62 | 0,65 | 26,9 | 1,04 | 16 | 17 | 195 | 31,9 | 9,8 | 14,4 | 0,414 |
| 35 | 13,8 | 4,75 | 88,9 | 32,9 | 29,4 | 270 | 8,1 | 8,75 | 1,41 | 6,65 | 0,61 | 27,1 | 1,07 | 17 | 18 | 186 | 29,7 | 12,4 | 20,4 | 0,284 |
| 36 | 13,3 | 4,58 | 90,7 | 32,5 | 29,4 | 272 | 7,7 | 8,02 | 1,38 | 6,05 | 0,56 | 27,4 | 1,05 | 18 | 16 | 198 | 27,8 | 13,8 | 20,9 | 0,215 |
| 37 | 15,2 | 4,65 | 95,1 | 34,4 | 32,7 | 249 | 7 | 9,16 | 2,22 | 5,84 | 0,69 | 26,6 | 0,85 | 34 | 88 | 253 | 52 | N/A | 59,2 | 0,93 |
| 38 | 12,7 | 4,22 | 94,1 | 31,9 | 30,8 | 340 | 7,2 | 6,1 | 1,4 | 4,08 | 0,33 | 17,4 | 1,05 | 19 | 21 | 189 | 18,6 | 47,9 | 116,7 | 0,13 |
| 39 | 13,9 | 4,43 | 94,5 | 32,5 | 31,4 | 292 | 7,4 | 8,19 | 2,26 | 4,85 | 0,49 | 21 | 0,52 | 24 | 50 | 219 | 32,8 | N/A | 70,2 | 0,133 |
| 40 | 14,4 | 4,75 | 86,6 | 34,9 | 30,2 | 274 | 7,8 | 8,15 | 1,17 | 6,47 | 0,37 | 28,7 | 1,2 | 19 | 18 | 172 | 26,7 | 29,7 | 42,2 | 1,041 |
| 41 | 14,1 | 4,86 | 85,6 | 33,9 | 29 | 205 | 11,8 | 9,49 | 2,42 | 5,54 | 1,23 | 23,7 | 0,98 | 29 | 46 | 225 | 35,2 | 56,3 | 60,9 | 0,815 |
| 42 | 13,6 | 4,52 | 89,7 | 33,1 | 30,2 | 274 | 8,4 | 7,26 | 2,31 | 4,65 | 0,61 | 22,4 | 0,91 | 21 | 33 | 202 | 28,4 | 38,9 | 72,7 | 0,272 |
| 43 | 13,8 | 4,68 | 92,4 | 32,8 | 28,9 | 239 | 10,2 | 8,37 | 1,59 | 5,86 | 0,92 | 23,2 | 0,96 | 25 | 38 | 213 | 31,6 | 50,2 | 70,8 | 0,19 |
| 44 | 13,9 | 4,62 | 89,8 | 33,4 | 29,8 | 254 | 9,11 | 8,28 | 1,96 | 5,48 | 0,84 | 23,5 | 0,91 | 24 | 37 | 205 | 30,9 | N/A | 46,7 | 0,014 |
| 45 | 10,57 | 3,57 | 89,7 | 33,5 | 30 | 294 | 7,9 | 8,14 | 1,14 | 6,24 | 0,57 | 23,9 | 1,01 | 20 | 12 | 214 | 18,2 | N/A | 78 | 0,455 |

Abb: Urea - urea level; Volume - tumor volume; Age – patient’s age; MCHC - mean corpuscularhemoglobin concentration; MYO18A - Myosin 18A; APTT - activated partial thromboplastin time; INR - international normalised ratio; MCH - mean cell hemoglobin; MCV - mean cell volume; PLT - platelets; PFS – progression-free survival; OS - overall survival; LYM - lymphocytes; RBC - red blood cells; CHOL - cholesterol level; WBC - white blood cells; NEU - neutrohils; Hb - hemoglobin; MONO - monocytes; MPV - mean platelet volume; AST - aspartate aminotransferase; ALT - alanine transaminase.


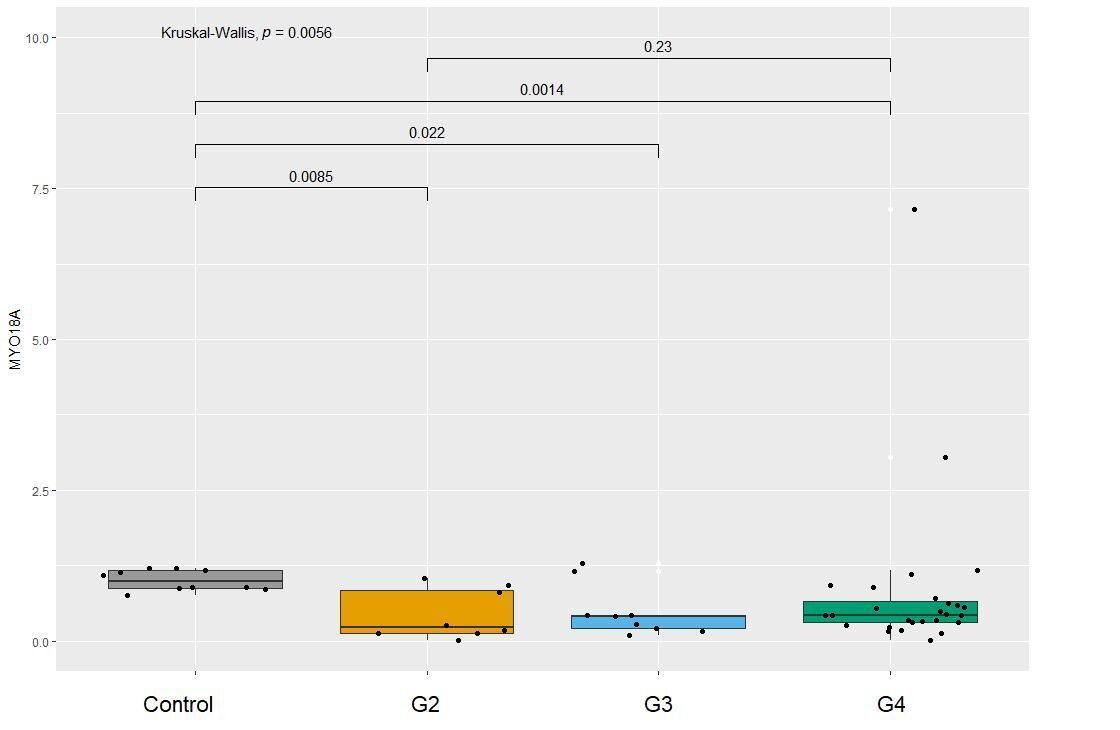


Figure S1. Differences in MYO18A RQ values between the control, G2, G3 and G4 group.


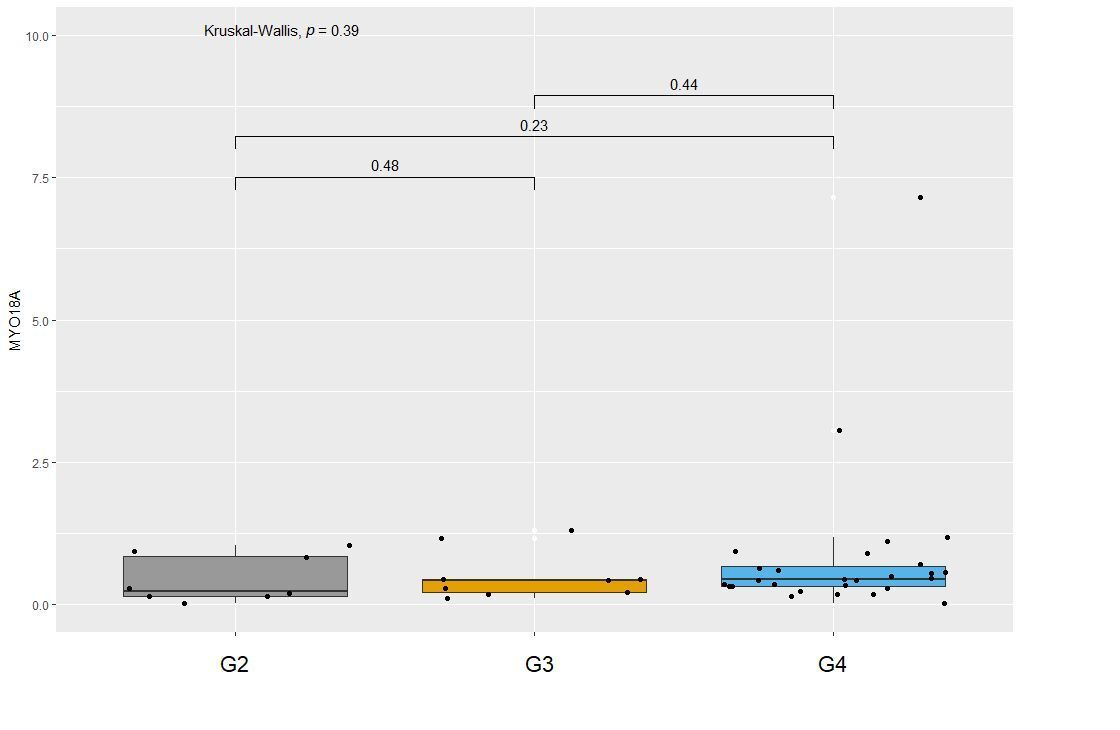


Figure S2. Differences in MYO18A RQ values between G2, G3 and G4 group.
